# Supplementary figures and images for: Early antiretroviral therapy and its impact on natural killer cell dynamics in HIV-1 infected men who have sex with men: a cross-sectional pilot study evaluating the impact of early ART initiation on NK cell perturbation in HIV infection
Source: Microbiol Spectr. 2024 Feb 16;12(4):e03570-23. doi: 10.1128/spectrum.03570-23 (PMC10986508; doi:10.1128/spectrum.03570-23)

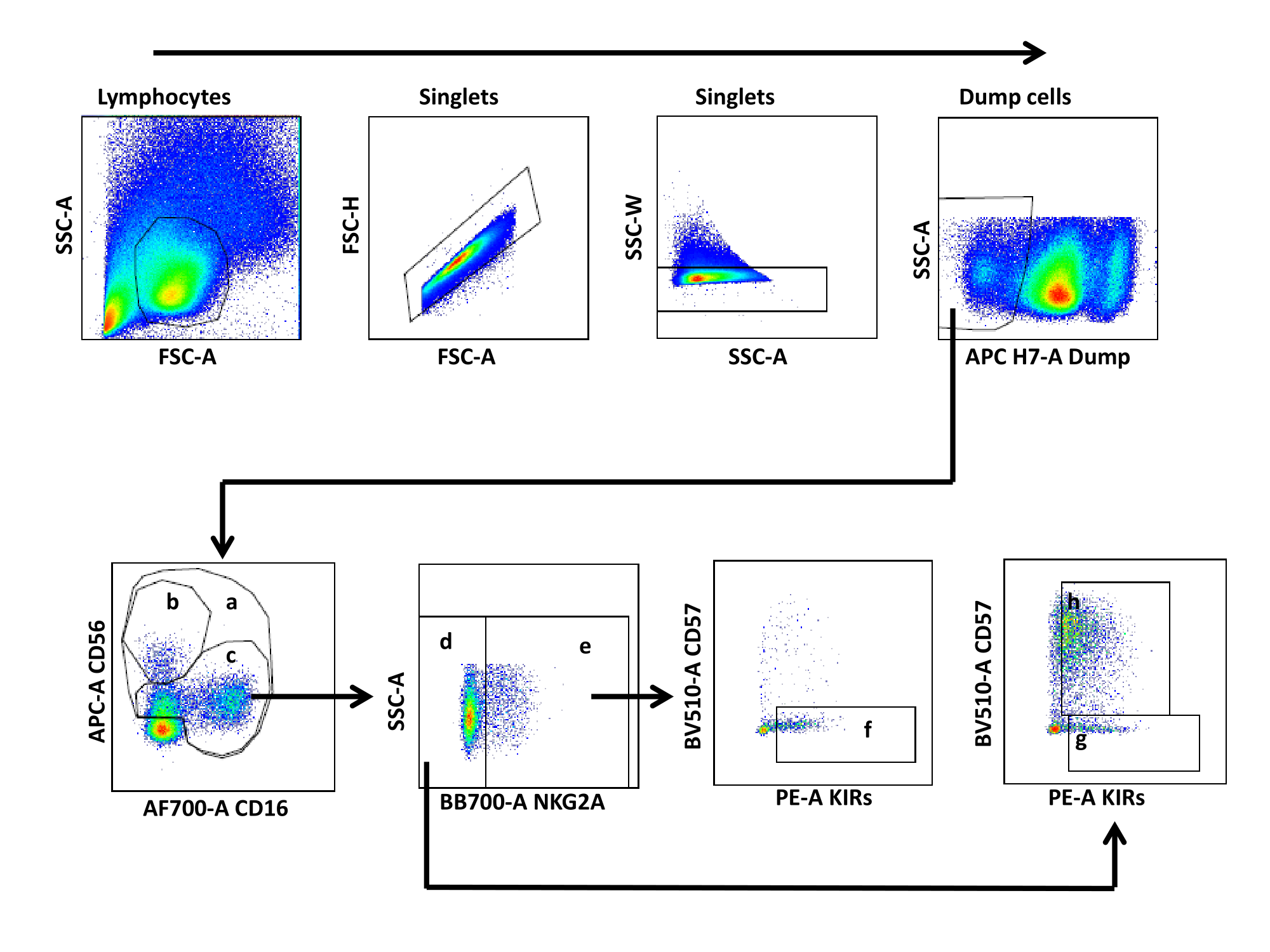

Supplement: Fig. S2 — Gating strategy for defining the different NK cell subsets. [file spectrum.03570-23-s0002.tiff]

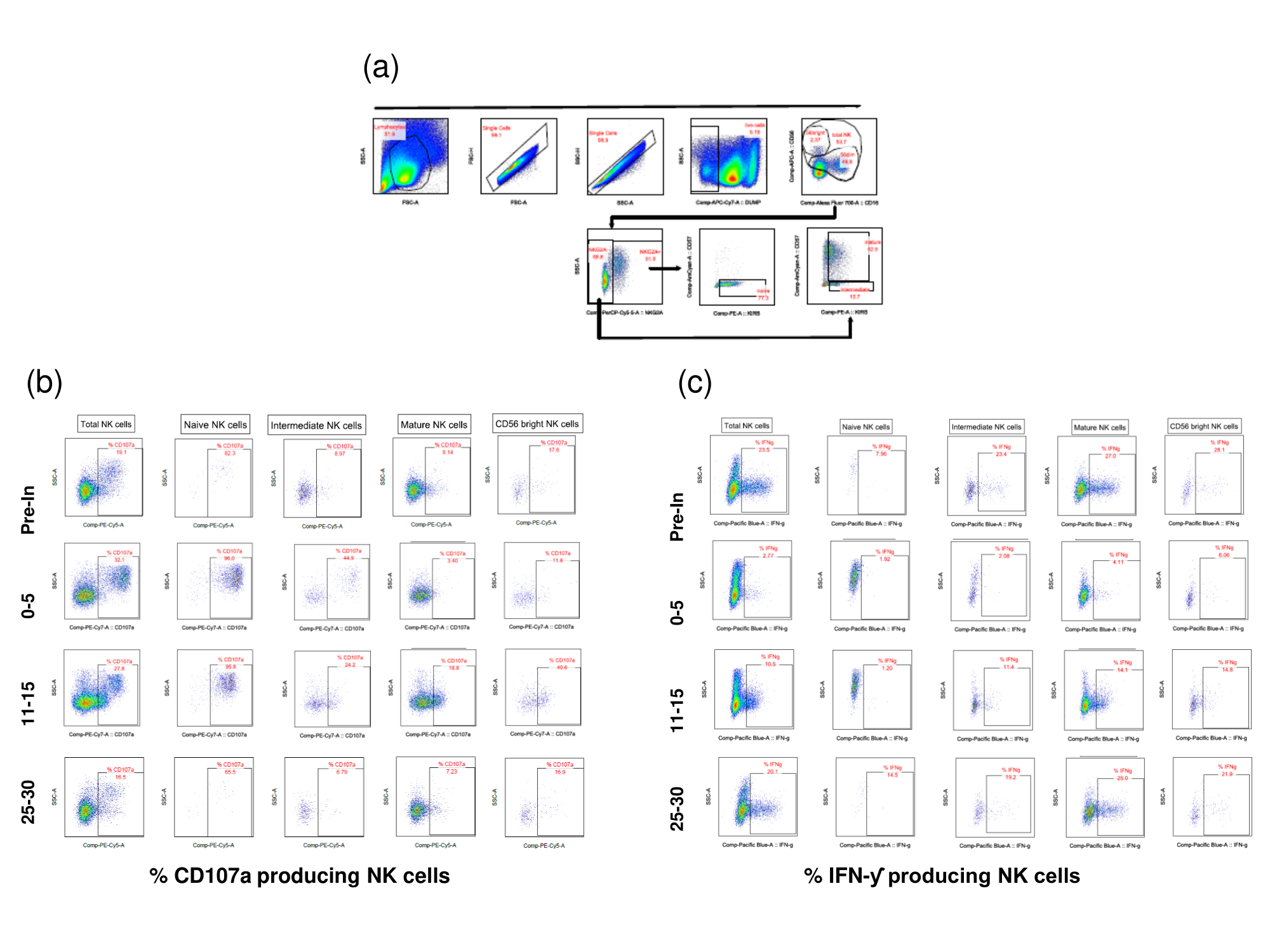

Supplement: Fig. S5 — Flow jo analysis and gating for participant NK02001 for the NK cell functional panel. [file spectrum.03570-23-s0005.tiff]
