# Supplementary material for: Early antiretroviral therapy and its impact on natural killer cell dynamics in HIV-1 infected men who have sex with men: a cross-sectional pilot study evaluating the impact of early ART initiation on NK cell perturbation in HIV infection
Source: Microbiol Spectr. 2024 Feb 16;12(4):e03570-23. doi: 10.1128/spectrum.03570-23 (PMC10986508; doi:10.1128/spectrum.03570-23)
Supplement: Legends — Titles for the supplemental figures and tables [file spectrum.03570-23-s0006.docx]

**Figure S1: Occurrences and changes in NK cell frequencies, CD4 cell counts and HIV viral loads across the different study time points.** (a) Series of events at different time points pre and post ART initiation, (b) changes in the frequencies of total NK cells from the total lymphocytes, (c) changes in absolute CD4 T cell counts and (d) changes in HIV-1 viral loads at different time points before and upon ART initiation. Statistical test used across time points: unmatched, non-parametric one-way ANOVA with multiple comparisons (mean of each column with the mean of a control column (pre-infection time point); Geisser-Greenhouse correction used). Statistical test used between time points: two tailed paired t test. Each symbol indicates a participant. Line indicates mean value. Error bars indicate standard deviation. Pre-In – pre infection (sample collected before the first seropositive test), ND – not done (test not done).

**Figure S2: Gating strategy for defining the different NK cell subsets.** (a) Total NK cells, (b) CD56bright NK cells, (c) CD56dim NK cells, (d) CD56dim NKG2A negative NK cells, (e) CD56dim NKG2A positive NK cells, (f) CD56dim naïve NK cells, (g) CD56dim intermediate NK cells and (h) CD56dim fully differentiated NK cells. The dump channel contained antibodies against CD3, CD14 and CD19.

**Figure S3:** **Frequency of total NK cells expressing phenotypic surface markers.** Cell activation markers CD38, CD69 and HLA-DR, cell exhaustion marker PD-1, NK cell activation marker NKG2C and NK cell inhibitory maker Siglec7 in pre-infection (pre-in) and in early HIV-1 infection at different time points upon ART initiation. Statistical test used between time points: two tailed paired t test. Statistical test used across time points: unmatched, non-parametric one way ANOVA with multiple comparisons (mean of each column with the mean of a control column (pre-infection time point); Geisser-Greenhouse correction used). Each symbol indicates a participant. Lines indicate paired samples from the same participant. *p<0.05, **p<0.01.

**Figure S4:** **Changes in the frequencies of functional NK cells at the different time points.** (a) Frequency of total NK cells producing IFN-ƴ and (b) frequency of total NK cells degranulating as denoted by surface expression of CD107a at pre-infection (pre-in) and at the different time points upon ART initiation. Statistical test used between time points: two tailed paired t test. Each symbol indicates a participant. Lines indicate paired samples from the same participant. *p<0.05.

**Figure S5:** **Flow jo analysis and gating for participant NK02001 for the NK cell functional panel**. (a) Defining the different NK cell subsets, (b) expression of CD107a by the different NK cell subsets at the different analyzed time points, (c) production of IFN-ƴ by the different NK cell subsets at the different analyzed time points.

**Table S1:** Demographic data of the study participants

**Table S2:** Anti-human antibodies used for flow cytometry staining
