# Supplementary material for: Early antiretroviral therapy and its impact on natural killer cell dynamics in HIV-1 infected men who have sex with men: a cross-sectional pilot study evaluating the impact of early ART initiation on NK cell perturbation in HIV infection
Source: Microbiol Spectr. 2024 Feb 16;12(4):e03570-23. doi: 10.1128/spectrum.03570-23 (PMC10986508; doi:10.1128/spectrum.03570-23)
Supplement: Additional experimental details — Detailed materials and methodology used in the study. [file spectrum.03570-23-s0007.docx]

**Supplementary material**

**Title:** Early Antiretroviral Therapy and its Impact on Natural Killer Cell Dynamics in HIV-1 infected Men who have sex with men: A cross-sectional pilot study evaluating the impact of early ART initiation on NK cell perturbation in HIV infection.

**Authors:** Matrona Akiso (MSc)^1, 4^, Robert Langat (PhD)^1,2^, Daniel Muema (PhD)^3,1^, Kewreshini K. Naidoo (PhD)^3^, Geoffrey Oino (MSc)^1^, Gaudensia Mutua (MD)^5^, Christina Thobakgale (PhD)^6,7^, Daniel Ochiel (PhD)^5^, Kundai Chinyenze (MD)^5^, Omu Anzala (PhD)^1,4^, Marianne Mureithi (PhD)^1,4^.

**Authors’ affiliation:**

^1^KAVI-Institute of Clinical Research, University of Nairobi, Kenya, ^2^Division of Surgical Outcomes and Precision Medicine Research, Department of Surgery, University of Minnesota Twin Cities, USA,^3^HIV Pathogenesis Programme, University of KwaZulu-Natal, South Africa, ^4^Department of Medical Microbiology and Immunology, University of Nairobi, Kenya, ^5^International AIDS Vaccine Initiative (IAVI). ^6^Faculty of Health Sciences, School of Pathology, University of Witwatersrand, South Africa, ^7^Centre for HIV and STI’s, National Institute for Communicable Diseases, Johannesburg, South Africa.

**Corresponding author:** Dr. Marianne Mureithi, P.O Box 19676-00202, Nairobi, Kenya. Mobile: +254703704711. E-mail: [marianne@uonbi.ac.ke](mailto:marianne@uonbi.ac.ke)

**Running title:** Early ART impacts NK cells in MSM

**Key words:** Acute HIV-1 Infection, ART, Innate immunity, Natural killer cells; MSMs, Seroconversion

**Materials and Methods**

**Study participants and ethical considerations**

This prospective study involved five (5) acutely HIV-1 infected MSM in Nairobi, Kenya. The study was approved by the Ethics and Review Committee of Kenyatta National Hospital, University of Nairobi (P61/02/2017). MSM aged 18 years and above, participating in the International AIDS Vaccine Initiative (IAVI)’s protocol Simulated Vaccine Efficacy Trial (SiVET: P137/03/2015) and HIV infected with laboratory-confirmed for anti-HIV antibodies using both the Alere Determine HIV 1/2 and Trinity Biotech unigold HIV 1/2 rapid kits were consented to participate in the study. The MSM were referred to a HIV counselling and testing centers (HCT) and immediately initiated on ART. Consented MSM were scheduled for the first (enrolment visit), when information on basic demographics, a brief medical history, physical examination, and concomitant medications were collected. This was followed by blood draws for hematology tests (Beckman Coulter AcT 5diff AL), CD4/CD8 counts (BD FACS count), viral load analysis (GeneXpert machine Dx system, Cepheid) and isolation of peripheral blood mononuclear cells (PBMCs). Blood samples were collected at two more visits scheduled at 2 weeks intervals for viral load, CD4/CD8 and PBMCs isolation.

**Processing of blood samples**

PBMCs were isolated as described previously (1). Briefly, approximately 20mls of whole blood was layered into 20mls of Histopaque (Sigma Aldrich, Co. St. Louis, MO USA) in a 50ml falcon tube for density gradient centrifugation at 1890 revolutions per minute (rpm) 40 minutes (min) without brakes (Multifuge 4 KR Heraeus). The plasma layer on top was then carefully harvested and placed in a clean 15ml falcon tube, leaving about half an inch of plasma in the 50ml falcon tube to avoid disturbing the PBMC layer. The harvested plasma was then centrifuged at 1800 rpm for 20 min with brakes to pellet any debris (Eppendorf Centrifuge 5804 R). The clear plasma was aliquoted into 1ml vials and stored at -80^o^C until use. The PBMCs were harvested carefully avoiding too much of the underneath Histopaque and placed into a clean 50ml falcon tube. Two washes with 40mls of Hankwo followed this washes with 40mls of Hankwo followed this washes with 40mls of Hanks followed this balanced salt solution (Sigma Aldrich Co. St. Louis, MO USA) and one wash with culture medium containing 20mls of RPMI (Sigma Aldrich, Co. St. Louis, MO USA) supplemented with 10% fetal bovine serum (FBS) (Sigma Aldrich, Co. St. Louis, MO USA), 1% 10000 units penicillin and 10mg/ml streptomycin (Sigma Aldrich, Co. St. Louis, MO USA), 1% 200 mM l-glutamine (Sigma life sciences), 1% 1M Hepes buffer (Sigma Life Sciences) and 1% 100 mM sodium pyruvate (Sigma Life Sciences), here referred to as ‘R10’. The cells were then counted in 5mls R10, and the cells were frozen at -198^o^C in freezing medium containing 90% FBS and 10% dimethyl sulfoxide (DMSO)(Sigma Aldrich, Co. St. Louis, MO USA) at a concentration of 10 million cells/ml per vial.

**Flow cytometric analysis**

PBMCs were thawed as previously described (1). The cells were then counted manually on a neubauer chamber using a microscope and resuspended at 10 million viable cells/mL in R10. Due to limited fluorochrome channels in our flow cytometer machines (BD LSR II), flow cytometry assay was done in two panels (activation panel for phenotypic and immune activation analysis of the NK cells and a functional panel for analysis of the NK cell functionality). For the functional panel, the cells were plated at 1 x 10^6^cells per well of a round bottom 96 well plate and stimulated with 1 x 10^5^ K562 cells line, 10ng interleukin (IL)-15 and 100U IL-2 per well. To each well, 3.5µl of CD107a-PECy7 (BD Pharmingen), 1µl of Brefeldin A (1mg/mL) and 1µl of Golgi stop was added and cells incubated at 37°C, 5% CO_2_ overnight (approximately 18 hrs). The following day, cells were centrifuged at 2000rpm for 10 minutes and washed once with 1X PBS; phosphate buffered saline (; Sigma Aldrich, Co. St. Louis, MO USA). The cells were then stained for viability with 100µl of fixable viability stain 780 diluted at 1:1000 in 1X PBS for 20 min at room temperature (RT) in the dark. This was followed by one wash with 1X PBS and then surface stained with a cocktail of antibodies (see supplementary table 1 for antibody details) for 20 min at RT in the dark. The cells were washed once with PBS followed by fixation and permeabilization with BD fixation/permeabilization solution for 20 min on ice in the dark. This was followed by a one wash with BD perm wash buffer (10X perm wash buffer was diluted to 1X with sterile filtered water prior to use (Sigma Aldrich, Co. St. Louis, MO USA)) and then intracellularly stained for IFN-ƴ using mouse anti-human IFN-ƴ BV421 (Biolegend) for 20 min at RT in the dark. The cells were washed twice with PBS, and resuspended in 200µL of PBS and data were acquired using a BD LSR II flow cytometer. For the activation panel, 1 x 10^6^ cells (from PBMCs at thaw) were plated in a round bottom well of a 96 well plate and stained with antibodies as shown in supplementary table 2.

**Defining the NK cell subsets**

To identify NK cells in the flow cytometry analysis, T cells, monocytes and B cells were excluded using a dump channel consisting of CD3, CD14 and CD19 respectively. The total NK cells (Figure S2a) were defined as the sum of CD56^bright^CD16- (Figure S2b) and CD56^dim^CD16-/+ cells (Figure S2c). From the CD56^dim^CD16-/+ NK cells, three sub populations were defined as: CD56^dim^ naïve NK cells (NKG2A+CD57-KIRS+) (Figure S2f), CD56^dim^ intermediate NK cells (NKG2A-CD57-KIRS+) (Figure S2g), and CD56^dim^ fully differentiated NK cells (NKG2A-CD57+ KIRS+) (Figure S2h). CD56^dim^CD16-/+NKG2A- and CD56^dim^CD16-/+NKG2A+ are shown in figure S2d and figure S2e respectively.

**HIV-1 viral load analysis**

HIV-1 viral load analysis was performed using a GeneXpert machine (GeneXpert machine Dx system, Cepheid) according to the manufacturer’s instructions. Briefly, the plasma was thawed to room temperature, and then 1 ml added into HIV-1 viral load cartridge and loaded into the GeneXpert machine.

**Data analysis**

Flow cytometry data were analyzed using Flow Jo software (version 10.8.1). Statistical analysis was performed using Graph Pad Prism software (version 8.0.1). Comparison between time points was done using a two tailed paired t test while comparisons among more than two time points was done using unmatched, non-parametric one-way ANOVA with multiple comparisons (mean of each column with the mean of a control column (pre-infection time point); Geisser-Greenhouse correction used). The cut off point for the level of significance of the results was calculated at a 95% confidence interval with a two-sided alpha value of 0.05 (p ≤ 0.05). Levels of significance are denoted here as; ns – not significant, * < 0.05, ** < 0.01, *** < 0.001.

**Ethical approval**

This study was approved by the Ethics and Review Committee of Kenyatta National Hospital and University of Nairobi (KNH/UoN/ERC) and assigned protocol number P61/02/2017.

**Availability of data and materials**

All relevant data generated in this study are available within the paper and its supporting information files at KAVI-ICR and can be obtained from the corresponding author on reasonable request.

1. Langat RK, Farah B, Indangasi J, Ogola S, Omosa-Manyonyi G, Anzala O, et al. Performance of international aids vaccine initiative african clinical research laboratories in standardised elispot and peripheral blood mononuclear cell processing in support of HIV vaccine clinical trials. Afr J Lab Med. 2021;10(1):1–13.
