# Supplementary material for: Early antiretroviral therapy and its impact on natural killer cell dynamics in HIV-1 infected men who have sex with men: a cross-sectional pilot study evaluating the impact of early ART initiation on NK cell perturbation in HIV infection
Source: Microbiol Spectr. 2024 Feb 16;12(4):e03570-23. doi: 10.1128/spectrum.03570-23 (PMC10986508; doi:10.1128/spectrum.03570-23)
Supplement: Table S1 — Demographic data of the study participants. [file spectrum.03570-23-s0008.pdf]

**Number**	**5**
**Sex**	**Male**
**Median age in years (IQR)**	**30(21-32)**
**Number (proportion) immediately initiated on ART**	**5(100%)**
**Median days post estimated date of infection (IQR)**	**78(50-81)**
