# Supplementary material for: Early antiretroviral therapy and its impact on natural killer cell dynamics in HIV-1 infected men who have sex with men: a cross-sectional pilot study evaluating the impact of early ART initiation on NK cell perturbation in HIV infection
Source: Microbiol Spectr. 2024 Feb 16;12(4):e03570-23. doi: 10.1128/spectrum.03570-23 (PMC10986508; doi:10.1128/spectrum.03570-23)
Supplement: Table S2 — Anti-human antibodies used for flow cytometry staining. [file spectrum.03570-23-s0009.pdf]

**Antibodies used in both the activation and functional panels**			
Anti-human CD3	Mouse	APC H7	BD Pharmingen
Anti-human CD14	Mouse	APC H7	BD Pharmingen
Anti-human CD19	Mouse	APC H7	BD Pharmingen
Anti-human CD16	Mouse	AF700	BD Pharmingen
Anti-human CD56	Mouse	APC	BD Pharmingen
**Antibodies used in the activation panel only**			
Anti-human HLA-DR	Mouse	PE-CY7	BD Pharmingen
Anti-human CD38	Mouse	PECF594	BD Horizon
Anti-human PD-1	Mouse	FITC	BD Pharmingen
Anti-human CD69	Mouse	BV421	BD Horizon
Anti-human NKG2C	Mouse	PE	R&D Systems
**Antibodies used in the functional panel**			
Anti-human NKG2A	Mouse	BB700	BD OptiBuild
Anti-human CD158a	Mouse	PE	BD Pharmingen
Anti-human CD158b	Mouse	PE	BD Pharmingen
Anti-human CD158e1/e2	Mouse	PE	Beckmen Coulter
Anti-human siglec7	Mouse	AF488	R&D Systems
Anti-human CD57	Mouse	BV510	Biolegend
Antihuman CD107a	Mouse	PE-Cy7	BD Pharmingen
Antihuman IFN-γ	Mouse	BV421	Biolegend
